# RabbitSketch: a high-performance sketching library for genome analysis

**DOI:** 10.1093/bioinformatics/btaf249

**Published:** 2025-04-26

**Authors:** Tong Zhang, Zekun Yin, Xiaoming Xu, Lifeng Yan, Fangjin Zhu, Xiaohui Duan, Bertil Schmidt, Weiguo Liu

**Affiliations:** School of Software, Shandong University, Jinan 250101, China; School of Software, Shandong University, Jinan 250101, China; School of Software, Shandong University, Jinan 250101, China; School of Software, Shandong University, Jinan 250101, China; School of Software, Shandong University, Jinan 250101, China; School of Software, Shandong University, Jinan 250101, China; Institute for Computer Science, Johannes Gutenberg University, Mainz 55128, Germany; School of Software, Shandong University, Jinan 250101, China

## Abstract

**Summary:**

We present RabbitSketch, a highly optimized library of sketching algorithms such as MinHash, OrderMinHash, and HyperLogLog that can exploit the power of modern multi-core CPUs. It provides significant speedups compared to existing implementations, ranging from 2.30× to 49.55×, as well as flexible and easy-to-use interfaces for both Python and C++. As a result, the similarity analysis of 455GB genomic data can be completed in only 5 minutes using RabbitSketch with merely 20 lines of Python code. As a case study, we enhanced RabbitTClust by integrating RabbitSketch’s Kssd algorithm, resulting in a 1.54× speedup with no loss in accuracy.

**Availability and implementation:**

RabbitSketch is available at https://github.com/RabbitBio/RabbitSketch with an archived version at Zenodo: https://doi.org/10.5281/zenodo.14903962. Detailed API documentation is available at https://rabbitsketch.readthedocs.io/en/latest.

## 1 Introduction

The rapid advancement of high-throughput technologies has led to a significant expansion of genomic sequencing data, posing huge challenges for data processing in bioinformatics (Rowe 2019). Even when taking advantage of high-performance multi- or many-core platforms, traditional algorithms often find it difficult to process large-scale genome data in practical time (Schmidt *et al.* 2024), creating computational bottlenecks that limit downstream research (Elworth *et al.* 2020). Thus, there is an urgent need for efficient data processing methods to manage genomic data at scale (Yin *et al.* 2017). Sketch algorithms, represented by the pioneering work of Mash (Ondov *et al.* 2016), have emerged as pivotal solutions for large-scale genome analysis in recent years. These algorithms transform raw sequence data into sketches, which are compact summaries of the original data, significantly reducing computational and storage costs. As the scale of genomic data continues to grow, sketch algorithms provide an increasingly effective tool.

Consequently, a variety of different sketching algorithms has been proposed. Popular examples in genomics include MinHash, OrderMinHash, Kssd, and HyperLogLog. MinHash generates sketches by hashing *k*-mers and retaining those with the smallest hash values. It has two primary implementations: K-Hash Function (KHF), which computes the minimum hash value for each hash function, and K Minimum Hash Values (KMV), which selects the top *k* smallest hash values using a single hash function. OrderMinHash (Marçais *et al.* 2019) enhances MinHash by introducing ordering of *k*-mers, considering their positional information and serving as a proxy for edit distance but is more complex as it requires multiple hashing rounds similar to KHF. Kssd (Yi *et al.* 2021) selects a subset of positions by randomly shuffling the total *k*-mer substring space and dividing it into *N* sub-spaces, where *N* denotes the dimensionality reduction level. HyperLogLog can estimate the cardinality of large datasets with limited memory consumption. Tools like Dashing (Baker and Langmead 2019, 2023) use HyperLogLog to generate sketches and estimate set cardinality by counting leading zeros in hash values.

Although various sketch algorithms have been proposed and applied to large-scale genomic data analysis, there are still several challenges in practice. First, there is no ready-to-use sketching library for the widely used high-level Python or C++ APIs. This lack forces developers to repeatedly implement the same algorithms, resulting in a significant waste of time and effort. Second, while sketching was originally designed for performance, existing implementations often do not fully leverage the features of modern processors, leading to slow runtimes. Moreover, despite the variety of available approaches, users cannot easily switch between algorithms without needing to rewrite code. This motivates the design of a comprehensive and high-performance sketch library.

To address this need, we present RabbitSketch, aiming to provide an efficient, flexible, and user-friendly sketch library. By featuring optimization for modern hardware and providing comprehensive support for the most commonly used algorithms, it shows superior efficiency and usability compared to existing implementations.

## 2 Methods

To address diverse requirements and ensure practical applicability (see Supplementary Section 1.2), we have selected four sketching algorithms that are widely used for genome sequence analysis, including MinHash, OrderMinHash, Kssd, and HyperLogLog.

### 2.1 MinHash

In our MinHash implementation, we use a branch-free method (Zhang *et al.* 2023) for computing reverse complement sequences, based on bitwise operations and table lookups, as shown in Supplementary Fig. 1a. This method is applied in all subsequent algorithms. When building sketches, we use the efficient KMV method, similar to other MinHash implementations (e.g. Mash). Unfortunately, we still face performance bottlenecks in hash computation and data manipulation. Therefore, we introduce SIMD-based implementations of string based (e.g., MurMurHash3 used in MinHash sketch building) and integer-based (e.g., Wang hash) hash functions (Yin *et al.* 2021). MurMurHash3 is optimized with both AVX2 and AVX512 instruction sets to process multiple data blocks in a single call, improving performance. We also reorganize input data layout to support this method as shown in Supplementary Fig. 1b. Additionally, we have introduced a faster and more memory-efficient robin-hood hashmap to enhance the performance of sketch data structure manipulation.

**Figure 1.**
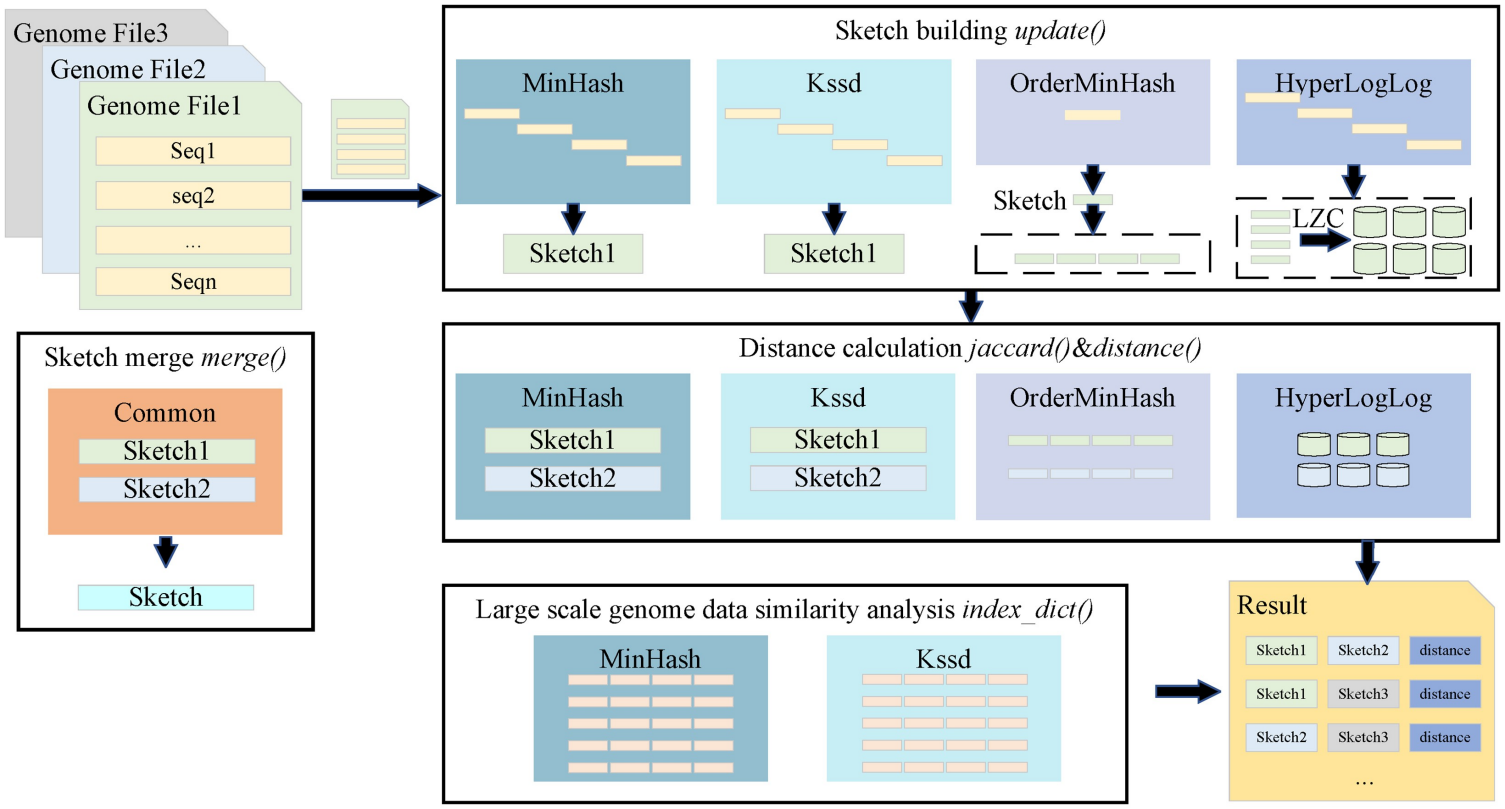
Example workflow of four algorithms in RabbitSketch. Except for OrderMinHash, all other algorithms support streamed building of sketches. The API for building sketches in all four algorithms is named update. The library also includes API for computing the Jaccard index and distance for different algorithms. Additionally, we provide an index_dict method designed to support large-scale genomic similarity analysis.

For distance computation, we reorganize the code according to our previous work (Yin *et al.* 2021) and adopt the block-based SIMD intersection method (Inoue *et al.* 2014) to reduce branch mispredictions in set intersections. Each block contains multiple elements from the sets.

### 2.2 OrderMinHash

During the sketch-building phase, OrderMinHash uses *m* hash functions and retains *l* minimum hash values for each function. Supplementary Fig. 1c shows the computation results for each hash function. A priority-queue method is used to keep track of the smallest *k* hash values, and the SIMD-based hash function addresses the computation bottleneck. For example, four *k*-mers are hashed simultaneously, as shown by the red box in Supplementary Fig. 1c.

Performance profiling indicates that after this optimization, the performance bottleneck shifts from computation to memory access, primarily due to repeated *k*-mer loading from the main memory. To address this issue, we have designed a cache-friendly data-reuse strategy. Instead of computing hash values row by row, we change the computation direction to column by column to achieve effective data reuse. In this direction, *m* hash value computations only require loading *k*-mer once from main memory, avoiding repeated memory access. However, this method requires maintaining *m* priority queues, which can be inefficient. To reduce overhead, we adopt a block-based method that takes advantage of data reuse while limiting the number of priority queues maintained simultaneously. The green box with an arrow in Supplementary Fig. 1c illustrates an example where 2×4 elements are packed into a block, and hash values are computed block by block in the horizontal direction. We can benefit from data reuse while limiting the overhead caused by maintaining multiple queues.

### 2.3 Kssd

The main hotspot in building Kssd sketches is retrieving the shuffle dictionary to find *k*-mers used for constructing sketches. Initially, the *k*-mers are hashed into integer hash values. These hash values are then retrieved from the shuffle dictionary to determine whether the hash values are valid for building the sketch. The shuffle dictionary is formed by shuffling the selected codes within the range of 0 to $2^{4\times \mathrm{space}}-1$. The $\frac{1}{2^{4\times d}}$ selected codes are regarded as valid codes, which means only $\frac{1}{2^{4\times d}}$  *o*f k-mers are valid hash values used for building sketches, where *d* is the dimension reduction parameter *drlevel*. However, only $\frac{1}{2^{4\times d}}$ of the hash values in the shuffle dictionary are valid, with the majority of the shuffle dictionary consisting of invalid hash values. Retrieving all *k*-mers from the shuffle dictionary to determine the valid hash values causes significant memory access bottleneck. To address this issue, we create a map dictionary that only includes the valid hash values using a robin-hood hashmap, where its size is $\frac{1}{2^{4\times d}}$ of the shuffle dictionary. Since the map dictionary is significantly smaller than the shuffle dictionary, it allows for much smaller retrieval latencies and can be stored in cache.

Similar to MinHash, the Kssd sketches only contain a set of shuffled hashes. We can simply compute the Jaccard index by counting the matched elements in two sketches based on the set intersection operation. Thus, we can directly adopt the block-based SIMD intersection method to improve the performance of distance computation, as described in Section MinHash.

### 2.4 HyperLogLog

Our implementation of HyperLogLog is based on the highly optimized Dashing framework (Baker and Langmead 2019), with additional performance enhancements. We employ SIMD hash functions to improve the efficiency during hash calculations. Moreover, on modern AVX512-enabled CPUs (supporting avx512cd and avx512vl instruction sets), we utilize SIMD *lzcnt* instructions to count the leading zeros of the hashes during sketch building. In comparing HyperLogLog sketches, determining the match status of corresponding buckets is essential. To mitigate overhead from branch mispredictions during sketch comparisons, we introduce a simple but effective branch-free method.

### 2.5 Extension to large-scale analysis

One of the most important problems in large-scale genome analysis is computing the pairwise distances between a large number of genomes. Popular applications like Mash, Dashing, and Kssd all support this function. Kssd introduced an optimal way called “index_dict” for similarity estimation. We extend this method to pure hash sketch methods (the sketch only includes a set of hashes) in RabbitSketch (see Supplementary Section 1.3 for details). However, the “index_dict” has many empty indices and high memory usage for large hash spaces. To address both issues, we can use a compact hashmap-based “index_dict” to avoid recording the empty indices (Xu *et al.* 2023a). Additionally, we recommend thread binding and memory replication strategies for NUMA architectures. These strategies minimize cross-node memory accesses, as illustrated in Supplementary Fig. 1d.

### 2.6 Library design

RabbitSketch provides a number of commonly used interfaces for sketch building and distance calculation. For sketch building, we provide both streaming and non-streaming interfaces. As for distance calculation, various interfaces for similarity estimation, such as reporting the Jaccard index or the Mash distance, are included. RabbitSketch also provides interfaces for other tasks, such as “merge” interface for building a single sketch based on a large number of sequences. Considering that Python has a broad user base, we also provide fully functional Python interfaces. The example workflow can be seen in Fig. 1. Additionally, RabbitSketch supports user-defined parameters for each algorithm, including *k*-mer size and sketch size. This enables researchers to adjust them according to their specific needs. More details can be seen in Supplementary Section 1.

## 3 Results

We have compared RabbitSketch to the latest versions of Mash (v2.3), OrderMinHash (v0.0.2), Kssd (v2.2.1), and Dashing (v1.0.2) on a 64-core Intel server supporting AVX512 and a 128-core AMD server supporting AVX2, both running Linux. In addition, we use the NCBI RefSeq Release 211 bacterial genome dataset as our experimental data. The detailed experimental setups can be seen in Supplementary Section 3. Specifically, we denote RabbitSketch algorithms with an “R” prefix to distinguish them from the original algorithms. Our performance evaluation is conducted on both the sketch generation process (sketch) and the distance calculation between sketches (dist). The total processing time (total) represents the sum of the times for the above two operations, indicating the overall time taken for analyzing genome sequence similarity.

We have implemented MinHash, Kssd, and HyperLogLog genome analysis tools using the C++ interfaces of the RabbitSketch library. OpenMP is used for multi-threading to compute all-to-all distances among the RefSeq bacteria dataset. We have compared the performance with existing implementations. During the experiment, a sketch is built regarding all sequences within each file, except for OrderMinHash. OrderMinHash, which proxies edit distance and considers relative order information, makes it impractical to compute a single sketch for an entire file with multiple sequences. As shown in Table 1, the optimized implementation in RabbitSketch achieves speedups ranging from 2.72× to 44.77× compared to the original implementation on the AMD server For MinHash, our implementation achieves a 3.14× overall speedup, with 1.19× and 8.14× speedups for sketch and distance operations, respectively, thanks to the block-based SIMD intersection method. We also present a case study to evaluate the efficiency of MinHash using the index_dict method (see Supplementary Section 4.2). Kssd implementation achieves a 6.61× overall speedup and 2.22× and 88.50× for sketch and distance operations, respectively, through efficient memory access strategies and a compact robin-hood hashmap. HyperLogLog achieves a 2.72× overall speedup and 5.23× for distance operations, with sketch building limited by I/O bandwidth. We also implement the multi-threading version of OrderMinHash, which takes only 7 s to process the SubRefSeq with 50 threads, achieving a two-orders-of-magnitude performance improvement compared to OrderMinHash. We have evaluated the thread scalability on this platform. RabbitSketch demonstrates linear speedup up to at least 32 threads, indicating effective thread scalability (see Supplementary Section 4.3). We have also tested the performance of RabbitSketch on an AVX512-enabled Intel server. Similarly, RabbitSketch achieves speedups of about 2.30–49.55× of the overall runtimes compared to the existing implementations, as shown in Table 1.

**Table 1.** Performance of RabbitSketch on the AMD and Intel server.

	Algorithm		Mash	RMash	OMH	ROMH	Kssd	RKssd	HLL	RHLL
AMD server.	Time (s).	sketch	353.552	296.974	5310.006	118.566	418.871	156.429	314.462	315.896
		dist	946.528	116.236	0.472	0.048	891.528	41.59	1142.026	217.973
		total	1300.080	413.210	5310.478	118.614	1310.399	198.019	1456.488	533.869
	speedup(total)		3.14		44.77		6.62		2.72	
Intel server.	Time (s).	sketch	171.776	158.043	5012.768	101.125	169.177	63.944	63.901	232.374
		dist	1410.346	211.856	0.314	0.040	303.698	32.852	1186.573	310.250
		total	1582.122	392.189	5013.082	101.165	472.875	96.796	1250.474	542.624
	Speedup(total)		4.03		49.55		4.88		2.30	

To evaluate the performance of our Python interface, we compared it with datasketch which is a popular sketch algorithm library implemented in Python. The results (see Supplementary Section 4.1 for details) show that compared to the popular Python-based sketch algorithms, RabbitSketch achieves high processing efficiency, achieving speedups from 43.20× to 99.32×, and has comparable efficiency with the high-performance C++ version. The similarity analysis of 455GB of genomic data can be completed within 5 minutes using RabbitSketch.

As a case study, RabbitTClust (Xu *et al.* 2023b) can complete the sketch building process effectively using the MinHash algorithm of RabbitSketch. This demonstrates the practical value of RabbitSketch in enhancing real-world applications. Furthermore, we improved RabbitTClust by replacing its original sketch algorithm with the Kssd algorithm from the RabbitSketch library. As a result, RabbitTClust achieved a 2.46× speedup in the sketch construction stage. It also achieved a 1.39× speedup in the whole process, with no loss in accuracy (see Supplementary Section 4.4).

## 4 Conclusion

In this article, we present RabbitSketch, an optimized sketching library for genome analysis. Through comprehensive experiments, we have demonstrated that it is effective in large-scale genomic analysis and surpasses previous high-performance implementations. RabbitSketch highlights significant advantages in both usability and efficiency, establishing itself as an important tool for downstream large-scale genomic analysis. Additionally, our provided Python interfaces greatly expand its applicability to accelerate real-world applications. As part of our future work, we plan to expand RabbitSketch based on the needs of the users, such as integrating an even larger variety of sketching algorithms and accelerating compute-intensive operations using GPU devices to provide even higher speeds.

## Supplementary Material

btaf249_Supplementary_Data

## Data Availability

The data underlying this article are available in the NCBI Reference Sequence Database release 211 at ftp://ftp.ncbi.nlm.nih.gov/refseq/release
