# Supplementary material for: RabbitSketch: a high-performance sketching library for genome analysis
Source: Bioinformatics. 2025 Apr 26;41(5):btaf249. doi: 10.1093/bioinformatics/btaf249 (PMC12054975; doi:10.1093/bioinformatics/btaf249)
Supplement: btaf249_Supplementary_Data [file btaf249_supplementary_data.pdf]

RabbitSketch supplementary

1 Library design

1.1 Overview

RabbitSketch provides a number of commonly used interfaces for sketch building and distance calculation. For sketch building, we provide both streaming and non-streaming interfaces. As for distance calculation, various interfaces for similarity estimation, such as reporting the Jaccard index or the Mash distance are included. We provide detailed explanations in the following subsections.

RabbitSketch also provides interfaces for other tasks, e.g., "merge" sketches. Considering that Python has a broad user base, it also features fully functional Python interfaces. Additionally, we support user-defined parameters for each algorithm, including k-mer size and sketch size. This enables researchers to adjust them according to their specific needs. We have now made our code available on multiple platforms:

Github:https://github.com/RabbitBio/RabbitSketch
BioConda: rabbitsketch
Zendo: https://doi.org/10.5281/zenodo.14903962
Figshare: https://doi.org/10.6084/m9.figshare.28562438
Software Heritag: swh:1:dir:37a2e92029166297bc46866a0b0de566d051385f

1.2 Algorithms of RabbitSketch

To address diverse requirements and ensure practical applicability, we have selected several sketching algorithms that are widely used sketching algorithms for biological sequence analysis. As a result, RabbitSketch integrates the following algorithms.

We initially selected MinHash and HyperLogLog when building the RabbitSketch algorithm library. MinHash is effective for approximate set similarity, used in deduplication, retrieval, and clustering tasks. HyperLogLog is known for its space efficiency in cardinality estimation for streaming data. Both algorithms have been widely used in bioinformatics and large-scale data processing, demonstrating their effectiveness and practical value (Rowe, 2019).

However, we recognize that MinHash and HyperLogLog do not preserve the relative ordering of k-mers, which is critical for applications requiring higher stability and accuracy. To address this, we integrate OrderMinHash, which not only captures k-mer content but also considers their relative order, thereby improving sensitivity to sequence structure. This approach bridges the gap between MinHash-based similarity estimation and edit distance(Marçais *et al.*, 2019), making it more suitable for applications that require order-aware comparisons.

We observe that MinHash uses a fixed-size sketch, which may negatively affect distance estimation when comparing datasets with significant size differences. Additionally, due to the many-to-one mapping of hash functions, MinHash may introduce hash collisions, affecting the stability and accuracy of similarity calculations. Therefore, we integrate Kssd to improve computational precision and enhance adaptability to datasets of varying sizes. Kssd has proved its practical value through adoption in various applications, such as Kssdtree (Yang *et al.*, 2024) and Metakssd (Yi *et al.*, 2024).

1.3 APIs of RabbitSketch

We offer both Python and C++ interfaces with the same names. The Python interface is implemented using the open-source library *pybind11*. The descriptions and functionalities of the major interfaces are shown in Table 1.

Among the four algorithms mentioned above, all except OrderMinHash support streaming sketch update (e.g., building a sketch using multiple sequences), thus, they all require "update" interfaces. This is because the core feature of OrderMinHash is preserving the positional information of elements, unlike other sketching algorithms, such as MinHash, which ignore the order. When trying to build an OrderMinHash sketch of multiple sequences, it is unable to determine the positional information across these sequences. Thus, it is only able to create independent sketches for each sequence. Therefore, OrderMinHash cannot support streaming operations when building a sketch based on multiple sequences.

We provide the "index_dict" API for MinHash ("index_tridist_MinHash") and Kssd ("index_tridist") to support large-scale genome similarity analysis. The "index_dict" method is specifically designed for large-scale genome similarity analysis. It calculates similarity by building an index dictionary to efficiently track hash collisions. Due to its lower computational complexity, the "index_dict" method is recommended for large-scale genome similarity analysis. For smaller-scale analysis, we recommend using the *distance* method, as it avoids the overhead of index dictionary building.

The "index_dict" method was first introduced by Kssd for pure-hash sketch methods, where the sketch contains only a set of hash values. However, HyperLogLog does not store hash values, but instead maintains a compressed

Table 1: RabbitSketch algorithm interfaces

Algorithm	C++&Python API	Type	Description
MinHash	update	low-level	Build and update sketch, supporting streaming processing
	jaccard	low-level	Calculate and return the Jaccard index to another sketch
	distance	low-level	Calculate and return Mash distance to another sketch
	loadMinHashes	low-level	Load hash values from files
	merge	low-level	Merge two sketches
	parameter configuration interfaces	low-level	Interface for setting relevant parameters
	index_tridist_MinHash	high-level	large-scale genome analysis
OrderMinHash	buildSketch	low-level	Build sketch
	similarity	low-level	Calculate and return similarity to another sketch
	distance	low-level	Calculate and return edit distance to another sketch
	parameter configuration interfaces	low-level	Interface for setting relevant parameters
Kssd	update	low-level	Build and update sketch, supporting streaming processing
	jaccard	low-level	Calculate and return the Jaccard index to another sketch
	distance	low-level	Calculate and return the Mash distance to another sketch
	parameter configuration interfaces	low-level	Interface for setting relevant parameters
	index_tridist	high-level	large-scale genome analysis
HyperLogLog	update	low-level	Build and update sketch, supporting streaming processing
	jaccard	low-level	Calculate and return the Jaccard index to another sketch
	distance	low-level	Calculate and return the Jaccard distance to another sketch
	merge	low-level	Merge two sketches
	parameter configuration interfaces	low-level	Interface for setting relevant parameters

probabilistic representation using registers. As a result, HyperLogLog is not compatible with this approach. In future work, we plan to explore similarity analysis methods that better align with HyperLogLog. As for OrderMinHash, its similarity calculation is based on edit distance, which is based on sequence order. But the "index_dict" method does not consider k-mer order, it only focuses on the overlap between two sets. This makes "index_dict" also unsuitable for OrderMinHash.

Additionally, we provide a "merge" interface when building sketches. When building a single sketch based on a large number of sequences (e.g., building a sketch based multiple genomes of a species), the input genomes can be divided into several blocks, and multiple sketch objects can be constructed in parallel according to the blocks. Then these sketches can be simply merged to one target sketch using the "merge" interface. Once all blocks are processed, the final sketch result can be obtained through the merge operation.

Furthermore, RabbitSketch provides highly tuned vectorized string-based (e.g., MurMurHash3 and xxhash) and integer hashing (e.g., Wang hash and thomas_mueller_hash) implementations.

These APIs can be divided in two types: low-level and high-level. Low-level APIs refer to smaller kernel APIs with focused, granular functions, such as "update" and "distance", while high-level APIs are more comprehensive and capable of implementing standalone functionalities, such as "index_dict". Low-level APIs are more flexible due to their independent and modular functionality. To maintain this characteristic, we intentionally refrain from implementing multithreading within these APIs. For high-level APIs, we have implemented multithreading internally to achieve optimal performance. A dedicated parameter is provided to allow users to specify the number of threads.

1.4 Parameters of RabbitSketch

We have predefined a set of default parameters to meet the need of typical use cases. Detailed parameter settings, including the parameters for the four sketch algorithms, are shown in Table 2.

The four considered algorithms all employ the k-mer sliding window strategy, making the k-mer size significant for accuracy. Based on research from relevant literature, the selection of k-mer size should keep a balance between sensitivity and minimizing random collisions. We set the k-mer size limit to 32 and recommend that during the use of RabbitSketch, the k-mer size for nucleotide sequences be set to 21.

A distinctive feature is that Kssd differs from other algorithms by defining *halfK* instead of directly specifying the size of k-mers. Experimental verification has shown that setting *halfK* to 10 (equivalent to a k-mer size of 20) can reduce random matching between k-mers, thereby enhancing the efficiency and

Table 2: Key parameters and descriptions of sketching algorithms

Algorithm	Parameter	Description
MinHash	k-mer	Size of k-mer, default 21, not exceeding 32
	sketchsize	Size of sketch, default 1000
	reverse	Whether to process reverse complement sequences (nucleic acid sequences), default TRUE
	Seed	Hash seed value,default 42
HyperLogLog	k-mer	Size of k-mer, default 21, not exceeding 32
	np	Bit pattern segmentation point, default 20,not exceeding 24
	reverse	Whether to process reverse complement sequences (nucleic acid sequences), default TRUE
	estim	The method of estimating set cardinality, currently supporting four types
Kssd	halfK	Half size of k-mer, default 10, not exceeding 16
	drlevel	Dimensionality reduction level,default 6
	space	Space allocated for shuffling dictionary, default 3
	reverse	Whether to process reverse complement sequences (nucleic acid sequences), default TRUE
OrderMinHash	k-mer	Size of k-mer,default 21, not exceeding 32
	l	Number of minimum hash values to retain, typically 2-5,default 2
	m	Number of hash functions, positively correlated with accuracy, default 500
	reverse	Whether to process reverse complement sequences (nucleic acid sequences), default TRUE
	seed	Hash seed value

performance of the algorithm while maintaining its accuracy.

Another key parameter is the size of the sketch. The MinHash algorithm allows direct specification of sketch size through the *sketchsize* parameter, while the other three methods determine the sketch size through a combination of parameters. For instance, the sketch size for the OrderMinHash algorithm is determined by two parameters, denoted as l and m. The parameter l determines the retention of the minimum hash values for each hash function, and the parameter m specifies the number of hash functions used. Therefore, the sketch size for the OrderMinHash algorithm is calculated as $l \times m$.

Additionally, when processing nucleotide sequences, due to the double-stranded structure of DNA sequences, treatment of the reverse complement strands is also required. Therefore, RabbitSketch includes parameters to specify whether to handle reverse complement sequences.

1.5 Sketch generation

We have implemented various sketch algorithms. For MinHash, the sketch is computed using the "update" interfaces in streaming mode. It first preprocesses the input sequence by calculating the sequence length and converting it to uppercase. Following this, the hash values of the k-mers (including the reverse complement) are calculated. The resulting hash values are inserted into a heap structure, which operates as a minimum heap to store and maintain the minimum values among the calculated hash values. Finally, the hash values within the minimum heap are converted into a list as the sketching result. Multiple MinHash sketches with the same parameters can be merged using the "merge" interface.

For Kssd sketching, firstly, a shuffled dictionary is created according to a spatially symmetrical kssp (k-mer substring selection pattern) by parameters of k-mer size and dimensionality reduction level. The shuffled dictionary divides the hash space into N parts and only one of the N parts is selected as the valid range, where N is determined by the dimensionality reduction level. All of the k-mers are generated from the sequence in a slide-window way, and the k-mers are encoded into the hash value with 2 bits per nucleotide. Only the k-mers with hash values in the valid range are selected to generate the sketches. The final bits of the hash value in the Kssd sketches are determined by both the k-mer size and the dimensionary level.

In the case of HyperLogLog, we initialize m buckets, with $m = 2^{np}$. Then, the first np bits of the hash value are considered as the bucket index. Finally, the corresponding value in the bucket is updated using the number of leading zeros in the remaining bits of the hash value to describe the data distribution.

The processes for sketch building described above are all packaged into interfaces named “update”, which support streaming sketch updates. This allows building sketches for multiple genomic sequences.

For OrderMinHash, we use a priority queue to maintain the l minimum values for each hash function and m hash functions are employed in total. Finally, the top l minimum hash values corresponding to substrings in each priority queue are concatenated to form the final sketch. OrderMinHash's sketch building process is implemented in the “buildSketch” interface.

1.6 Distance computation

In this subsection, we provide a brief overview of the implementation of the distance calculation interfaces.

For MinHash, we calculate the Jaccard index by comparing the MinHash sketches of two sequences. The Jaccard index is measured by computing the ratio of the intersection size to the union size of two sets. The Jaccard index (J) between two sets A and B is defined as Equation 1:

$$J(A,B) = \frac{|A \cap B|}{|A \cup B|} \tag{1}$$

In this formula: $|A \cap B|$ denotes the size of the intersection of sets A and B, $|A \cup B|$ denotes the size of the union of sets A and B. In MinHash, the degree of hash value overlap directly reflects the similarity between sequences.

For OrderMinHash, the similarity scores are computed by directly comparing the elements of the sketches in both forward and backward (if reverse complement is enabled) directions. Only the higher score of the two directions is taken as the similarity.

For Kssd, when estimating the distance between two genomes, we construct an index dictionary based on the generated sketches, and use this index dictionary to build an intersection matrix that counts the occurrences of shared hash values. Then the Jaccard and containment coefficient can be calculated according to the shared hash values.

For HyperLogLog, we estimate the number of elements in each bucket based on the maximum leading zero count, and then calculate the cardinality of a sketch using these estimated values. We implemented three methods for estimating cardinality: ORIGINAL, ERTL_MLE, and ERTL_JOINT . The ORIGINAL method uses a corrected harmonic mean for cardinality estimation, the ERTL_MLE method enhances accuracy by modeling register counts as Poisson variables, and the ERTL_JOINT method directly estimates set intersections by jointly estimating Poisson parameters for elements unique to each set and their intersection. Subsequently, RabbitSketch estimates the cardinality of their union and intersection and calculates the Jaccard index.

We provide various distance metrics such as Jaccard distance and Mash distance. The Jaccard distance is calculated by subtracting the Jaccard index from 1. The Mash distance is defined as Equation 2:

$$D_{\text{Mash}} = -\frac{1}{k} \log\left(\frac{2J}{1+J}\right) \tag{2}$$

2 Optimization of methods

We fully leverage the hardware architecture features of modern processors, and optimize memory access and computation based on the characteristics of different sketch algorithms. The typical strategies can be seen in the Figure 1 These include some general optimization techniques, such as SIMD hash functions and computing reverse complemen methods, as well as optimization strategies customized for the algorithms. Overall, these strategies accelerate the computation process and optimize memory access strategies.

3 Experimental setup

We have compared RabbitSketch to the latest versions of Mash(v2.3), OrderMinHash(v0.0.2), Kssd(v2.2.1) and Dashing(v1.0.2). Specifically, we denote RabbitSketch algorithms with an 'R' prefix to distinguish them from the original algorithms. Our performance evaluation is conducted on both the sketch generation process (sketch) and the distance calculation between sketches (dist). The total processing time (total) represents the sum of the times for the above two operations, indicating the overall time taken for analysing genome sequence similarity. Note that, parameters can have significant impact on performance.

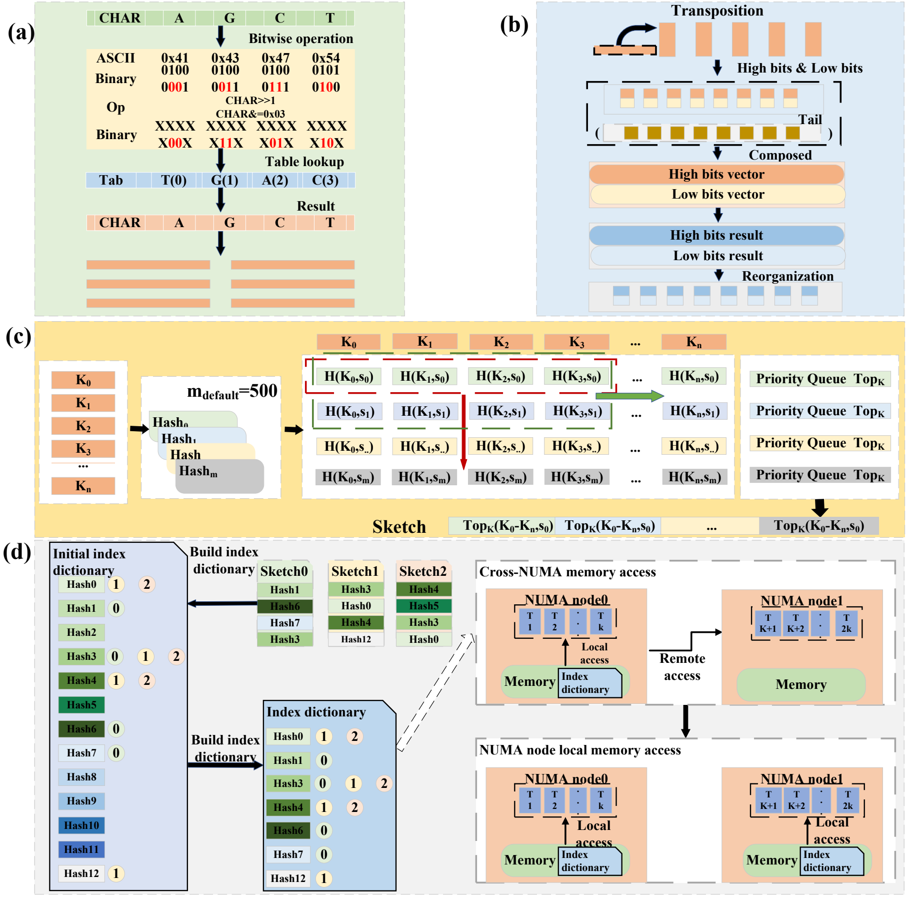

Figure 1: Typical strategies for algorithm optimization in the RabbitSketch library. (a) A branch-free method based on bitwise operations and table lookup techniques for computing reverse complement sequences. (b) The SIMD-based optimization of MurMurHash3. (c) Cache-friendly strategy for OrderMinHash. (d) Efficient memory access strategies for optimizing distance calculation

To be fair, we set the key parameters identical to the competing tools in the tests for each sketch algorithm, see Table 3.

Experiments are conducted on two platforms: an AMD server and an Intel server. The AMD server supports the AVX2 instruction set, while the Intel server supports the AVX512 instruction set. The details are provided in Table 4. In addition, we use the NCBI (National Center for Biotechnology Information) RefSeq Release 211 bacterial genome dataset as our experimental data. This dataset comprises 113,674 bacterial genomes, stored in FASTA format, totalling 455GB in size. The detailed information of this dataset is shown in Table 5. In

addition, the existing implementation of OrderMinHash lacks support for multi-threading and is extremely inefficient. We thus only choose a subset of RefSeq bacteria genome sequences to compare its performance with our implementation. It's important to note that, RMinHash uses an intersection-based method to compute all-to-all distances similar to Mash. Similarly, both RKssd and Kssd employ the *index dict* method.

4 Case study

4.1 Python performance evaluation

To evaluate the efficiency of the Python interface and account for fluctuations in runtime, we randomly sample 1000 genome sequences from the RefSeq dataset for testing(OrderMinHash still use the SubRefSeq). According to the results in Table 7, the extra time cost of using the Python interface can be ignored. The result proves our Python version of RabbitSketch has comparable efficiency with the high-performance C++ version.

To further evaluate the performance of our Python interface, we compared it with datasketch[1], which is a popular sketch algorithm library implemented in Python. The experimental results show that compared to the popular Python-based sketch algorithms, RabbitSketch achieves high processing efficiency, achieving speedups from 43.20x to 99.32x. As a case study, we have implemented a 20-line Python script using RabbitSketch and pymp (for multi-threading) libraries to build MinHash sketches. This script can generate sketches of the 455GB RefSeq bacteria dataset in about 6 minutes. The testing results demonstrate that our RabbitSketch library can effectively handle large-scale datasets using the Python interfaces.

4.2 MinHash-index_dict performance evaluation

We have present an efficient distance calculation method called *idnex_dict* in subsection 2.5. We proved the performance of Kssd through experiments in the Results section. Therefore, in this subsection, we provide a case study to demonstrate the effeciency of MinHash combined with this method for similarity analysis on large-scale datasets. The result can be seen in the Tab 6. Since we did not modify the sketches building phase, its runtime remains consistent. However, in the distance computation phase, the "index_dict" method significantly outperforms the pairwise comparison method.

[1] https://github.com/ekzhu/datasketch

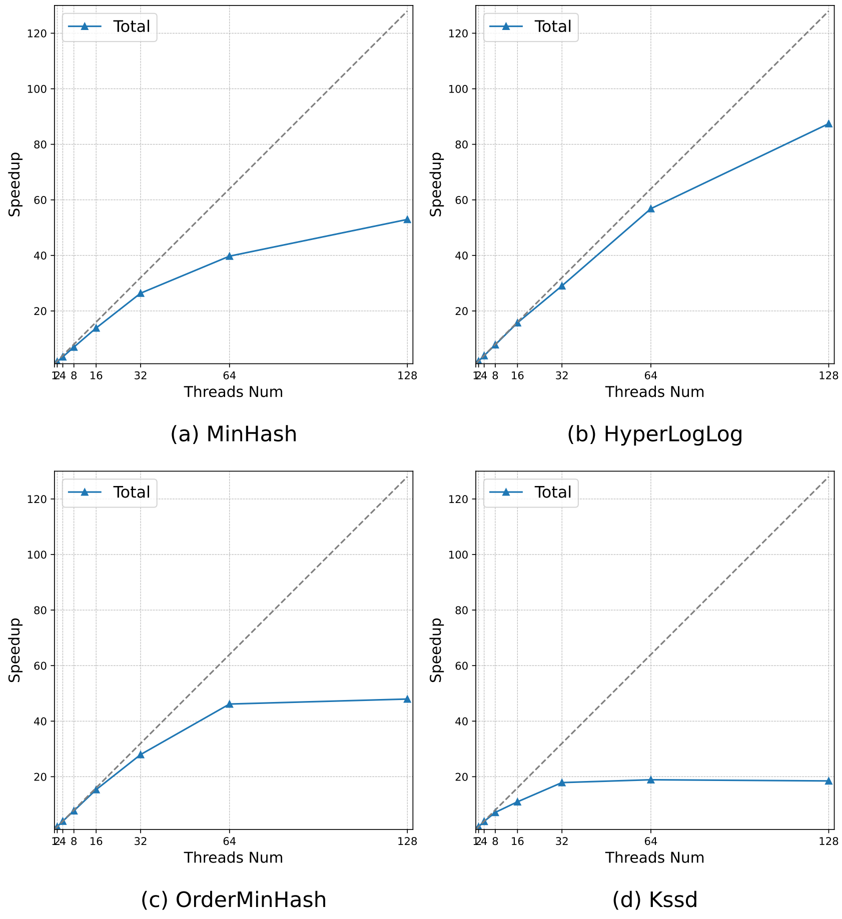

(a) MinHash (b) HyperLogLog

(c) OrderMinHash (d) Kssd

Figure 2: RabbitSketch algorithms' thread scalability on AMD servers

4.3 Thread scalability evaluation

Thread scalability is a key metric for evaluating RabbitSketch's performance. We have tested RabbitSketch on the AMD server across multiple threads to verify the thread scalability. The speedups of total processing using various threads are shown in Figure 2. When using less than 32 threads, most algorithms achieve a linear speedup. This is because the computational tasks and independent memory access tasks can be effectively distributed across multiple threads. As the number of threads increases, disk I/O bandwidth becomes the main limiting factor, making it difficult to significantly improve speed by adding more threads. Instead, I/O contention and thread scheduling overhead cause

the speedup to level off.

4.4 Integration of RabbitSketch to RabbitTClust

RabbitTClust is a tool for clustering large genomes. It uses the MinHash algorithm from the RabbitSketch library to build sketches and calculate similarity. As shown in Table 8, RabbitTClust built sketches for the 455GB bacteria dataset in 160 seconds on the Intel server. This reached the I/O peak of the workstation's SSD. This shows the practical value of RabbitSketch. We also replaced the MinHash algorithm with the Kssd algorithm from the RabbitSketch library. This achieved a 1.54× speedup in total. In terms of clustering accuracy, both methods achieved similar results. The clustering based on the MinHash strategy scored 0.961, and the clustering based on the Kssd strategy scored 0.960.

References

Marçais, G. *et al.* (2019). Locality-sensitive hashing for the edit distance. *Bioinformatics*, **35**(14), i127–i135.

Rowe, W. P. (2019). When the levee breaks: a practical guide to sketching algorithms for processing the flood of genomic data. *Genome biology*, **20**(1), 1–12.

Yang, H. *et al.* (2024). Kssdtree: an interactive python package for phylogenetic analysis based on sketching technique. *Bioinformatics*, **40**(10), btae566.

Yi, H. *et al.* (2024). Metakssd enables rapid and online metagenomic taxonomic profiling and profile searching. *bioRxiv*, pages 2024–06.

Table 3: Key parameter settings of RabbitSketch

Algorithm	Parameter	Value
MinHash	k-mer	21
	sketchsize	1000
	reverse	TRUE
OrderMinHash	k-mer	21
	l	2
	m	500
	reverse	TRUE
Kssd	halfK	10
	drlevel	3
	space	6
	reverse	TRUE
HyperLogLog	k-mer	21
	np	10
	reverse	TRUE

Table 4: Software and hardware configuration of the test platforms

Environment	AMD server	Intel server
CPU	AMD EPYC 7T83	Intel Xeon Platinum
Cores	128	64
NUMA nodes	2	2
Compiler	GCC 8.5.0	GCC 8.5.0
RAM	256G	256G
Storage	7.68T SSD	2T SSD * 4 (Raid0)

Table 5: Key Parameter Settings of RabbitSketch

Dataset	RefSeq	SubRefSeq
Data Source	NCBI	Subset of RefSeq
Release Number	211	211
Content	Bacteria genomes	Bacteria genomes
Genome Number	113,674	50
File Format	FASTA	FASTA
Total Data Size	455G	158MB

Table 6: Comparison of Mash-index_dict and Mash-pair_comp Methods

Method	Mash-index_dict	Mash-pair_comp
Dataset	RefSeq	
Environment	Intel server	
Time (sketch)	158.043	160.553
Time (dist)	211.856	180.826
Time (total)	392.189	341.379

Table 7: Performance evaluation of Python API

Algorithm	Version	Sketch(s)	Dist(s)
MinHash	RabbitSketch(C++)	72.392	0.610
	RabbitSketch(Python)	72.494	0.730
	DataSketch	>7200	-
Kssd	RabbitSketch(C++)	46.261	10.038
	RabbitSketch(Python)	47.130	10.152
	DataSketch	-	-
HyperLogLog	RabbitSketch(C++)	166.412	2.844
	RabbitSketch(Python)	166.652	3.061
	DataSketch	>7200	-
OrderMinHash	RabbitSketch(C++)	118.566	0.048
	RabbitSketch(Python)	119.542	0.051
	DataSketch	-	-

Table 8: Performance comparison of two sketch algorithms from the RabbitSketch library used by RabbitTClust

	Time (s)			Speedup		
	Sketch	Clustering	Total	Sketch	Clustering	Total
RabbitTClust-MinHash	160.069	540.258	700.327	-	-	-
RabbitTClust-Kssd	65.074	388.459	453.533	2.46	1.39	1.54
